# 

**Published:** 1901-02-23

**Authors:** 


### NOTICE TO CORRESPONDENTS.

[illegible] MSS., Letters, Books for Review, and other matters intended for the Editor should be addressed The Editor, "The Hospital," The Hospital Building, 28 & 29 Southampton Street, Strand, W.C.

The Editor cannot undertake to return rejected MS., even when accompanied by stamped directed envelope.

### Notice to Advertisers and Subscribers.

All Advertisements, Orders for Copies of The Hospital, and Business Communications should be addressed to **THE MANAGER ([illegible] to the Editor)**, The Hospital, 28 & 29 Southampton Street Strand, London, W.C.

The Cost of Subscription, post free, is as follows:—Per week, 2½d.; per quarter, 2s. 8d.; per half-year, 5s. 3d.; per annum, 10s. 6d. Foreign:—Per annum, 15s. 2d., payable, in all cases, in advance.

**NOTICE.—Vol. XXVIII. of "The Hospital" (April to September, 1900), in handsome cloth binding, is now on sale at the Office of this Journal, price, 7s. 6d. Cases for binding the Numbers contained in this Volume 1s. 6d. Index to Vol. XXVIII., 2½d., post free.**

**The Monthly Part for March will be ready on March 1st. Price 6d., by post 8d.**

### CRAPS AND GLEANINGS.

AT a meeting of the Manchester Infirmary Board a recommendation was confirmed to the effect that, as an experiment, "Kosher" food for Jewish in-patients should be introduced for one year under such restrictions as might be considered necessary.

THE report presented at the annual meeting of the Newcastle Lying-in Hospital showed that 178 patients had been attended at their dwellings and 32 in the hospital during the past year. The expenditure for the past year was £439 odd and the receipts £399.

THE new hospital buildings of St. John and St. Elizabeth, St. John's Wood are capable of accommodating 100 patients. The price paid for the site was £13,000, and the estimated cost of the new building is £45,000. The sum of £30,000 was received from the sale of the old building. A notable feature of the new building is the church, built by Sir G. Bowyer in Great Ormonde Street, which has been removed stone by stone and re-erected.

